# Experimental Crossing of Two Distinct Species of Leopard Geckos, *Eublepharis angramainyu* and *E*. *macularius*: Viability, Fertility and Phenotypic Variation of the Hybrids

**DOI:** 10.1371/journal.pone.0143630

**Published:** 2015-12-03

**Authors:** Jitka Jančúchová-Lásková, Eva Landová, Daniel Frynta

**Affiliations:** Department of Zoology, Faculty of Science, Charles University in Prague, Prague, Czech Republic; Fred Hutchinson Cancer Research Center, UNITED STATES

## Abstract

Hybridization between distinct species of animals and subsequent genetic introgression plays a considerable role in the speciation process and the emergence of adaptive characters. Fitness of between-species hybrids usually sharply decreases with the divergence time of the concerned species and the divergence depth, which still allows for a successful crossing differs among principal clades of vertebrates. Recently, a review of hybridization events among distinct lizard species revealed that lizards belong to vertebrates with a highly developed ability to hybridize. In spite of this, reliable reports of experimental hybridizations between genetically fairly divergent species are only exceptional. Here, we show the results of the crossing of two distinct allopatric species of eyelid geckos possessing temperature sex determination and lacking sex chromosomes: *Eublepharis macularius* distributed in Pakistan/Afghanistan area and *E*. *angramainyu*, which inhabits Mesopotamia and adjacent areas. We demonstrated that F_1_ hybrids were viable and fertile, and the introgression of *E*. *angramainyu* genes into the *E*. *macularius* genome can be enabled via a backcrossing. The examined hybrids (except those of the F_2_ generation) displayed neither malformations nor a reduced survival. Analyses of morphometric and coloration traits confirmed phenotypic distinctness of both parental species and their F_1_ hybrids. These findings contrast with long-term geographic and an evolutionary separation of the studied species. Thus, the occurrence of fertile hybrids of comparably divergent species, such as *E*. *angramainyu* and *E*. *macularius*, may also be expected in other taxa of squamates. This would violate the current estimates of species diversity in lizards.

## Introduction

The fact that related species of animals are sometimes able to hybridize is known since the beginning of evolutionary biology [1]. Nevertheless, the crucial importance of hybridization of animal species for evolutionary processes has been largely overlooked for decades (but see [2–6]). In recent years, molecular markers allowed zoologists to detect occurrence of natural between-species hybrids in the field. As a result, presence of hybrid zones and/or introgressed genes has been documented in many animal taxa (e.g., fruit flies: [7]; butterflies: [8]; fishes: [9]; toads: [10]; snakes: [11]; lizards: [12]; Darwin’s finches: [13]; nightingales: [14]; house mice: [15]; dolphins: [16]). This suggests that at least in the terminal branches of the phylogenetic tree, a predominantly divergent pattern of evolution caused by cladogenesis may be supplemented by a complementary process (syngenesis). This process breaks incomplete reproductive isolation mechanisms (RIMs) among related species, enabling genetic introgression from a donor species to a recipient one. The recipient populations may benefit from a gene flow supplying alien alleles. These effects on the fitness have been already tried and tested in the donor population. A recombination with the introgressed alleles can give rise to hopeful transgressive phenotypes with extreme trait values exceeding the combined range of parental species [4, 17–19]. Moreover to these evolutionary advantages, especially hybrids of the first filial generation and backcrosses, may improve their fitness due to overdominance and/or masking of the deleterious recessives, usually referred to as heterosis or hybrid vigour [20–24]. In extreme cases as, e.g., some of the Darwin’s finches, interspecific hybrids exhibit elevated fitness when compared with the parental species and genetic identities of the species have become fuzzy [25].

Hybrid sterility and/or inviability contribute fundamentally to reproductive isolation and delimitation of animal species. In a typical case, fitness of between-species and sometimes also between-population hybrids, especially those of F_2_ and other segregating generations, is considerably reduced. This phenomenon is referred to as an outbreeding depression [20, 26]. Dobzhansky (1936, 1937) [27, 28] and Muller (1940, 1942) [29, 30] recognized that the easiest way to the evolution of postzygotic reproductive isolation mechanisms (RIMs) of this kind is a genetic interaction (incompatibility) of alleles belonging to separate genes (loci). The original prevailing A_1_A_1_B_1_B_1_ genotype is replaced with A_2_A_2_B_2_B_2_ in the daughter population that becomes reproductively isolated due to reduced fitness of the hybrids (typically A_1_A_1_B_2_B_2_ and A_2_A_2_B_1_B_1_). Accumulation of Dobzhansky-Muller incompatibilities (DMIs) is probably a function of time that elapsed from the divergence of the crossed species [31]. This theoretical prediction was corroborated by experimental data in multiple taxa of animals (e.g., in frogs [32], in pigeons and doves [33], in centrarchid fishes [34], in *Drosophila* fruit fly [35, 36], in galliform birds [37], *Triturus* newt [38], but see [39] for the main role of sexual selection in hybridizing sword tail fishes). However, little is known about evolutionary rate at which these incompatibilities arise.

In vertebrates, hybrids of extremely distant genera were reported in fishes (e.g., Lepisosteidae: *Lepisosteus* and *Atractosteus* separated for 33–100 million years [40, 41]; Centrarchidae: *Acantharchus* and *Micropterus* separated for ~35 million years [34] and frogs (e.g., Hylidae: *Hyla* and *Pseudacris* separated for 22–80 million years [42, 43]). The time required for accumulation of efficient postzygotic RIMs varies considerably even among the principal clades of amniots (for details of genetic divergence in lizards, see the review [44]). The best documented comparison represents at least five-fold difference between mammals, typically loosing the ability to produce viable F_1_ hybrids after one or two million years of separation, and birds loosing this ability after 20 million years [45–47]. Divergence time estimates reported for marine turtles producing viable hybrids are even longer (e.g., *Chelonia x Caretta* [42], estimated to ~ 63 mye [48]). Vital and sometimes also fertile hybrids of distinct species/genera are also known for other chelonian taxa (e.g., Bataguridae: *Cyclemys x Occadia* [49]; *Mauremys x Saccalia* [50]; Chelidae: between some of the species in the genus *Chelodina* [51]). This may be attributed to a slow mutation rate reported in the chelonians [52]. The crocodylians, a sister taxon of the birds, are also able to produce viable between-species hybrids (e.g., *Crocodylus siamensis x C*. *rhombifer* [53]). Nevertheless, the genus *Crocodylus* is relatively young; the oldest records of this genus are known from the end of the Miocene [54]. In contrast to the high level of species diversity of lizards and snakes, there is only limited information about the time required to establish the postzygotic RIMs in most lineages of squamates. Most examples of viable F_1_ hybrids of squamates come from unisexual species (e.g., *Leiolepis* [55]; *Darevskia* [56]; *Aspidoscelis* [57]; *Lepidodactylus* [58]; *Hemidactylus* [59]; *Heteronotia* [60]; *Nactus* [61]). In these cases, however, further reproduction of the hybrids that may be otherwise sterile is enabled by parthenogenesis and/or multiplication of the gene dosage (triploidy, tetraploidy). Except for the parthenogens and their close relatives, also viable F_1_ hybrids of lizards belonging to distinct species or genera were reported in, e.g., true iguanids (e.g., *Conolophus x Amblyrhynchus* [62, 63]; *Ctenosaura similis x C*. *bakeri* [64]; *Iguana iguana x I*. *delicatissima* [65]) and lacertids (e.g., within the genus *Lacerta*: [66–70]; within the genus *Podarcis*: [71, 72]). Similar cases were repeatedly reported in snakes, e.g., pythons (*Morelia* x *Liasis* [73]; *Python natalensis* x *P*. *molurus bivittatus* [74]), colubrids (*Pituophis catenifer sayi x Pantherophis vulpinus* [75]) and viperids (*Vipera nikolskii* x *V*. *berus* [76, 77]). In our previous paper [44], we reviewed the available records of hybridization events in lizards and found that the upper limit of the HKY distance of cyt b gene between parental species producing viable homoploid bisexual hybrids is 19%; the corresponding distance for parental species of parthenogenetic hybrids is 21%. We also found that the experimental studies reliably reporting and documenting their further reproductive success in lizards are exceptional (but see [66–69], for a review see [44]).

The above mentioned differences among the higher taxa of amniots in the time-scale required for the evolution of postzygotic RIMs may have fundamental consequences on speciation patterns, which should be considered in the conservation theory and practice. The risk of outbreeding depression should be considered in defining taxonomic and/or population genetic delimitation of the conservation units in endangered species [78, 79]. Too broad definition of these units leads to a rapid increase in the expenses as well as demographic and genetic risks of extinction associated with small population numbers [78, 80–82].

In search of a dyad of model lizard species with allopatric distribution ranges that have been separated by well-dated geological events, we focused on the Middle East region. The Iranian Plateau and Zagros Mountains represent a distinct geographic barrier that limits the distribution and prevents contacts between lowland dwellers of Mesopotamia-Persian Gulf and those of Central Asia and Indian subcontinent [83]. History of these units is precisely known according to geological evidence; they originated as a result of a collision between Arabia and Eurasia plates that started 35–20 million years ago. Nevertheless, the main uplift of this area occurred 15–12 million years ago [84, 85]. Further topography growth of the external Zagros, Alborz, Kopet Dagh and Caucasus mountain belts reached its maximum 5 million years ago [86]. The long-lasting presence of the above described geographic barrier has clear consequences on a phylogenetic and phylogeographic structure of several reptilian taxa in Iran and adjacent areas; e.g., species complexes of the *Laudakia caucasica* [87, 88], *Eremias persica* [89], and *Mesalina watsonana* [90].

*Eublepharis macularius* (BLYTH, 1854), a lizard belonging to the family Eublepharidae, is a common laboratory animal, which is widely used as a model species of squamate reptiles in physiological [91–95], behavioural [96, 97], and evolutionary [98, 99] research. The distribution range of *E*. *macularius* includes large territories of Afghanistan, Pakistan and India [100]. Other species of the genus *Eublepharis* [100] are also distributed on the Indian subcontinent (*E*. *hardwicki*, *E*. *fuscus*) and Turkmenistan (*E*. *turkmenicus*). Another distinct species of the genus *Eublepharis*, the *E*. *angramainyu* (ANDERSON AND LEVITON, 1966) inhabits Mesopotamia and SW Iran [83]. The range of the *E*. *angramainyu* is separated from those of the *E*. *macularius* and remaining species of the genus *Eublepharis* by the Iranian Plateau and Zagros Mountains [100]. Thus, the dyad of the *E*. *angramainyu* and *E*. *macularius* represents a promising model of species that underwent a long-lasting geographical isolation.

One may argue that the seashore along the Gulf of Oman was penetrable for the geckos of the genus *Eublepharis* at least in the past. However, sequence divergences between mitochondrial genes of the *E*. *macularius* and *E*. *angramainyu* are considerable (uncorrected p-distances for 303 bp fragment of cyt b gene exceed 19%; HKY85 distance 22%, Palupčíková unpublished data) and fully congruent with the geological dates of the main uplift of the Iranian Plateau.

The aim of this paper is to examine the ability of distinct lizard species evolving separately for several million years to hybridize and exchange genes. For this purpose we crossed the *E*. *angramainyu* and *E*. *macularius* under laboratory conditions and assessed (1) viability, (2) fertility and (3) phenotypic characters (body size, body shape, coloration pattern) of the hybrids and parental species. Successful production of viable and fertile F_1_ crosses of our model species would further support the hypothesis that lizards possess slow (“avian” or “chelonian”) rather than rapid (“mammalian”) pattern of postzygotic RIM acquisition [44]. In accord with the general model of Dobzhansky-Muller incompatibilities and the empirical evidence in other animal taxa [101], we predicted that putative fitness losses affect more hybrids of F_2_ generation than those of F_1_ generation (all possessing a genotype A_1_A_2_B_1_B_2_).

## Materials and Methods

### Ethics Statement

All performed experiments were allowed by institutional Animal Care and Use Committee of the Charles University in Prague, and approved by Ethical Committee of Ministry of Education, Youth and Sports of the Czech Republic license no. 18147/203 and 24773/2008–10001. All animals from nature were purchased from a Czech company importing animals in the year 2002 and from private breeders. *Eublepharis* sp. does not belong to the species whose trade is limited by the CITES agreement or any other known regulations. According to the IUCN categorization it neither belongs to endangered species. After the study, geckos were used either for other behavioural experiments or for breeding purposes.

### Experimental procedures

The breeding stocks of the parental species were 38 females and ten males of an *E*. *macularius* (the first generation of descendants of wild-caught animals imported from Pakistan) and only five females and three males of the rare *E*. *angramainyu* (wild-caught animals and their two daughters; a putative locality of origin: Choqa Zanbil, Khuzestan province, Iran, 32"00'N 48'31'E, for more details about the locality see [102]).

To obtain F_1_ hybrids, 17 virgin females of the *E*. *macularius* were allowed to copulate with one breeding male of the *E*. *angramainyu*. The resulting F_1_ hybrids were reared to sexual maturity and further bred to obtain F_2_ hybrids and/or backcrosses with either *E*. *macularius* or with the same breeding male of the *E*. *angramainyu* (with their father). Fertility of some of the backcross hybrids was subsequently assessed by crossing with the parental species (for details see under the Results and Table 1). Because the geckos of the genus *Eublepharis* are able to store sperm for several months, each experimental female was allowed to copulate exclusively with a single male during a given mating season (lasting from January/February to July/August). In contrast, males were allowed to copulate with multiple females within a single breeding season. 15 F_1_ hybrid females were experimentally crossed for more than one breeding season; this allowed us to test their fertility with two or three different males (first with F_1_ male or one of the parental species and then with a male of the other parental species). As controls for the hybridization experiments, 16 females of the *E*. *macularius* and five *E*. *angramainyu* females were bred with conspecific unrelated males (with the exception of two *E*. *angramainyu* females, which were the daughters of the breeding male).

**Table 1:** The incubation success of eggs (hatchability) and survival rates of hatchlings. The parental species (*E*. *macularius—*P_M_, *E*. *angramainyu*—P_A_), their hybrids of the first (F_1_) and second (F_2_) filial generations, backcrosses of F_1_ females to male of *E*. *angramainyu* (B_1A_; denoted as MAxA), the reciprocal backcrosses of F_1_ males or females to *E*. *macularius* (B_1M_; the individuals with father F_1_ hybrid are denoted as MxMA, while those with mother F_1_ hybrid as MAxM), and two categories of higher order hybrids (crosses of MxMA females with males of either *E*. *macularius* or *E*. *angramainyu*). The above mentioned generations and/or crossings refer to the embryos and hatchlings.

Crossing abbreviation	M	A	MA	MAxMA		MAxA		MAxM		MxMA	(MxMA)xA	(MxMA)xM
**Mother**	P_M_	P_A_	P_M_	F_1_		F_1_		F_1_		P_M_	B_1M_	B_1M_
**Father**	P_M_	P_A_	P_A_	F_1_		P_A_		P_M_		F_1_	P_A_	P_M_
**Egg/hatchling**	P_M_	P_A_	F_1_	F_2_		B_1A_		B_1M_		B_1M_	B_1M_xP_A_	B_2M_
**No. of mothers**	16	5	17	13		10		22		10	3	2
**No. of clutches**	47	26	37	41		24		68		29	10	4
**No. of eggs**	90	42	71	81		42		131		57	17	7
**Temperature [°C]**	28	26	28	28	26	28	26	28	26	28	28	28
**No. of incubated eggs**	87	38	70	55	16	16	15	106	18	55	13	6
**No. of juveniles**	80	13	31	4	0	0	0	44	6	41	1	3
**Egg hatchability (%)**	92	34	44	6		-		40		75	8	50
**Survived to one year**	67	11	28	1		0		39		27	1	1
**Survival rate (%)**	84	85	90	25		-		78		66	100	33
**Sex ratio: Males/females**	9/58	4/7	3/25	0/1		0/0		4/35		2/25	0/1	0/1

The animals were housed individually in glass terrariums 60 x 30 x 20cm or 30 x 30 x 20cm in size. The ambient temperature in the breeding room was about 28°C with permanent presence of basking cables under every terrarium to maintain a temperature gradient. The floor of each cage was covered with bark substrate. Paper shelters, as well as feeding and drinking dishes, were provided. During the laying season, containers with adequately humid coconut substrate for egg deposition were added. The geckos had continuous access to water and were fed crickets and mealworms dusted with vitamins and minerals (Nutri Mix) weekly; AD_3_ and E vitamins were provided once per 14 days. The hatchlings were housed singly in plastic boxes 20 x 20 x 15cm and were fed solely the vitamins dusted crickets up to the three months of their age.

We studied the following nine categories of the parental species and their hybrids that are further referred to as follows (the abbreviations are given in parentheses; on the first place there is always an abbreviation for a female, then cross (x) with a male on the second position; the number and the sexes of these specimens in Table 1):

1. P_M_−the parental generation of the *E*. *macularius*, both parents belong to the *E*. *macularius* (M);
2. P_A_−the parental generation of the *E*. *angramainyu*, both parents belong to the *E*. *angramainyu* (A);
3. F_1_ –the first generation hybrid, a mother of the *E*. *macularius* and a father of the *E*. *angramainyu* (MA);
4. F_2_ –the second generation hybrid, both parents are F_1_ hybrids of the *E*. *macularius* and *E*. *angramainyu* (MAxMA);
5. B_1A_ –the first generation backcross with the *E*. *angramainyu*, a mother is an F_1_ hybrid and a father belongs to the *E*. *angramainyu* (MAxA);
6. B_1M_ –the first generation backcross with the *E*. *macularius*, a mother is an F_1_ hybrid and a father belongs to the *E*. *macularius* (MAxM);
7. B_1M_ –the first generation backcross with the *E*. *macularius* (reciprocal to 6), a mother belongs to the *E*. *macularius* and a father is an F_1_ hybrid (MxMA);
8. B_1M_ x P_A_−a higher order hybrid, a mother is the B_1M_ hybrid (cf. 7) and a father belongs to the *E*. *angramainyu* ((MxMA)xA);
9. B_2M_ –the second generation backcross with the *E*. *macularius*, a mother is the B_1M_ hybrid (cf. 7) and a father belongs to the *E*. *macularius* ((MxMA)xM).

Respective to the nocturnal activity pattern of the geckos and their thermal preferences [103], the mating attempts were conducted in the evening (after 7 p.m.) in a temperature-controlled breeding room (28°C) illuminated by a single red 25-W light bulb. Prior to the experiment, the females were weighed and were controlled for their receptivity by a visual inspection of the folicular growth through the abdomen wall [93]. We gently placed the male into the female’s terrarium for 30 min and we recorded the copulation behaviour using a night vision video camera. If mating did not occur within this interval, we repeated the trial the other day. The primary aim was to allow successful mating and to enable the production of fertilized eggs.

During the egg-laying season (since February to September), we controlled the egg-deposition containers for three times a week. The eggs were weighted and placed to the temperature-controlling incubator in plastic boxes, each containing a single clutch. We set the temperature to 28.5 ± 0.5°C, which is an optimal and preferred incubation temperature in the *E*. *macularius* [97, 104, 105]. Nevertheless, according to our previous experience with the *E*. *angramainyu*, the successful development of their embryos require slightly lower temperatures and longer incubation time. At 28°C incubation temperature (an upper limit for successful incubation), some hatchlings possessed a prolapsed yolk pouch. After consultation with other experienced colleagues at this field (e.g. Lukáš Kratochvíl, Charles University), we set the incubation temperature to 26 ± 0.5°C for the eggs laid by the *E*. *angramainyu*. The only feasible solution was to perform the experiments within the temperature range of 26–28°C, among which the incubation temperature overlaps in both species included in the experiment. Consequently, the eggs laid by the F_1_ hybrid females were initially incubated either at 26°C or at 28°C to compare the hybrid hatchability at the optimum incubation temperature for both parent species (at 26°C in *E*. *angramainyu* and at 28°C in *E*. *macularius*). The temperature was selected at random for the first clutch and then regularly switched in successive ones (see Table 1). In additional backcrossing of the F_1_ females with the *E*. *macularius* males in the breeding season 2013, which was aimed to prove their fertility, the incubation temperature was set to 28°C.

For every egg we took down the identity of the parents, the dates of laying and hatching, the weights of egg and hatchling and the incubation temperature. In order to perform formal tests of the hatchability, we used GLMs, in which the hatching of the incubated eggs of an individual clutch (number of hatchlings of one clutch and number of non-hatched eggs of the same clutch) was given as a dependent variable with binomial distribution and logit link function; the juvenile form, the incubation temperature and its interactions, and the clutch sequence were introduced as category explanatory variables. The calculations were performed in the R (R Development Core Team, Vienna, Austria).

Most eggs that have failed to hatch until the standard terms [106] were dissected to prove the presence and developmental stage of the embryos. Nevertheless, the content of many rotten eggs was entirely decayed, which precluded a reliable dissection. Thus, in many cases, we were unable to distinguish the fertilized eggs from those unfertilized.

The hatchlings were weighted and scanned (a ventral and a dorsal view of the body) in standardized positions. This procedure was repeated in adulthood at the age of 2–3 years. In order to provide a reference in the form additional fully grown individuals, the data set was supplemented with adult specimens of *E*. *macularius* from Pakistan and *E*. *angramainyu* from Iran (both wild-caught individuals and their descendants). In total, we collected 91 valid records for juveniles (*E*. *angramainyu*– 4 specimens, *E*. *macularius*– 32 spec., MA– 25 spec., MAxMA– 3 spec., MxMA– 11 spec., MAxM– 16 spec.) and 139 valid records for the animals older than two years (*E*. *angramainyu*– 10♀, 5♂, *E*. *macularius*– 55♀, 13♂), MA– 24♀, 3♂, MAxMA– 1♀, MxMA– 15♀, 2♂, MAxM– 7♀, 3♂, MMAxA– 1♀).

**Fig 1:**
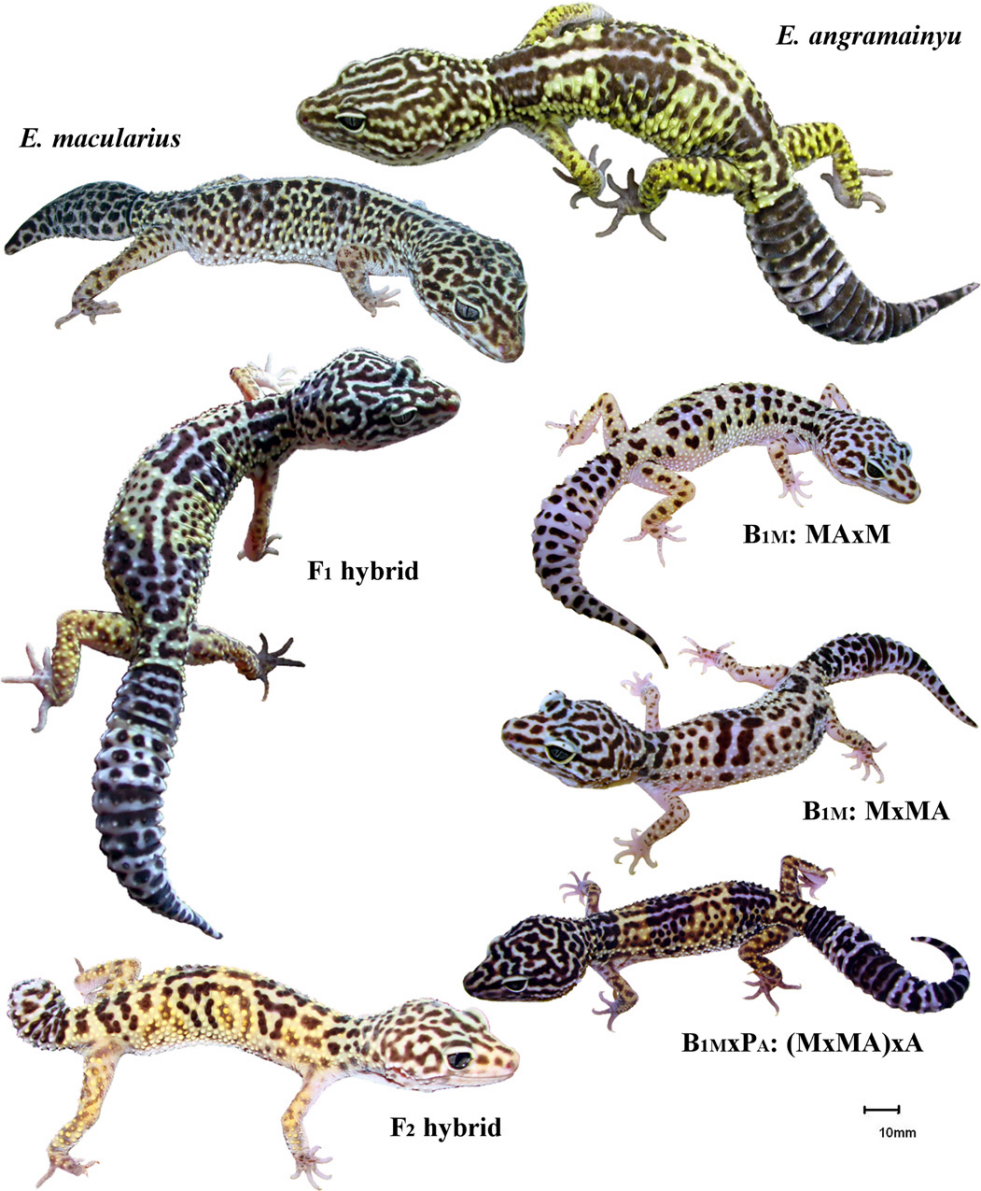
The external appearance and coloration. *E*. *macularius* (P_M_), *E*. *angramainyu* (P_A_), their hybrid of the first (F_1_) and second filial generations (F_2_), backcrosses of the F_1_ with male or female *E*. *macularius* (B_1M_: MAxM and B_1M_: MxMA, respectively), and a cross between a female of the latter backcross and a male of the *E*. *angramainyu* (B_1M_xP_A_). The scale bar used was 10mm.

**Fig 2:**
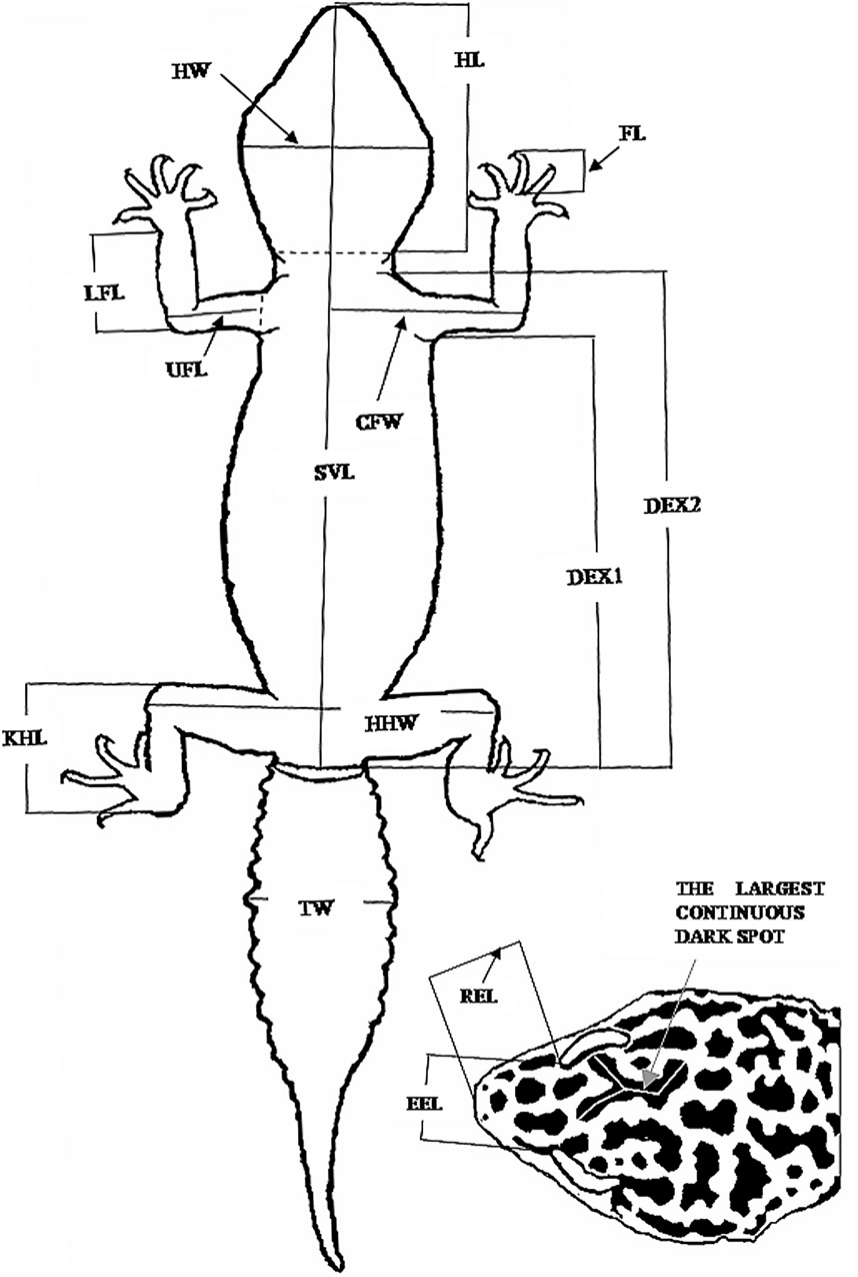
Measurements of the body and the head. SVL: snout-vent length; DEX1: from the margin of the front leg to the cloacal lips; DEX2: from the margin of collar to the cloacal lips; TW: tail width; UFL: upper fore-limb length; CFW: chest and upper fore-limb width; LFL: lower fore-limb length; FL: finger length; HHW: hip upper hind-limb width; KHL: knee to heel length; HL: head length; HW: head width; EEL: length between eyes; REL: rostrum to eye length; the largest spot: length and area was measured; the number of spots was computed.

The coloration pattern analysis of the *E*. *angramainyu* (29 spec.), *E*. *macularius* (29 spec.), F_1_ (28 spec.) and the B_1M_ (27 spec.) hybrids we conducted on a dorsal view of the head. For this purpose, we examined the scans of the animals older than one year with fully developed adult coloration pattern (Fig 1, also in [97]) First, the scans were set to black and white colors (converted to Grayscale mode, then to Bitmap mode by 50% Threshold method in Adobe Photoshop CS2; Adobe Systems Incorporated, USA). The total number of dark (melanistic) spots and the length of the longest continuous spot were performed by UTHSCSA Image Tool (San Antonio, Texas). The area of the largest continuous dark spot was measured in ImageJ program (National Institutes of Health, USA) (Fig 2). All measurements were calibrated using a squared paper present in each scan.

To test the effect of species/hybrid category on the adult coloration pattern on the head, we analyzed the Number of spots (square-root transformed), Spot size (area of the largest spot scaled to the head size and natural log-transformed) and Spot length (length of the largest spot scaled to the head length and natural log-transformed) using linear models with the form of the animal (P_M_, P_A_, F_1_, B_1M_) as a factor. Post hoc Tukey tests were adopted to compare the factor levels. The calculations were performed using STATISTICA, version 6.0 (StatSoft Inc., Tulsa, USA).

For morphometric analyses we adopted and/or modified standard measurements from Kratochvíl et al. (2003) [107] and Frýdlová et al. (2011) [108]. We used the following 14 measurements that were measured by UTHSCSA Image Tool from digital images: (1) SVL–snout-vent length; (2) DEX1 –distance between the extremities (from the posterior margin of the front leg to the cloacal lips); (3) DEX2 –from the posterior margin of collar to the cloacal lips; (4) TW–tail width (the largest width of the tail); (5) UFL–upper fore-limb length; (6) CFW–chest and upper fore-limb width; (7) LFL–lower fore-limb length (without hand); (8) FL–middle finger length without the claw; (9) HHW–hip upper hind-limb width; (10) KHL–knee to heel length; (11) HL–head length (from rostrum to the posterior margin of collar); (12) HW–head width, the largest width of the head; (13) EEL–distance between anterior corners of eyes; (14) REL–rostrum to eye length, from tip of the snout to the anterior corner of eye. In case of juveniles we measured only SVL. For the definition of these measurements, see Fig 2.

In order to separate a shape component of the morphometric variation, we performed the size-adjustment of the original variables. For this purpose, we used the method published by Somers (1986, 1989) [109, 110] as implemented in the Size analysis v02 [111–113]. This software computes not only generalized (multivariate) isometric size of the original untransformed measurements, but also partial isometric size-adjusted measurements. These size-free data were further analyzed by a multivariate exploratory statistics as implemented in the discriminant function analysis (DFA) subroutine of STATISTICA, version 6.0. The data were checked for normality prior to the statistical analyses. Deviations from normality were small, and most distributions were both unimodal and symmetrical as required for the used multivariate procedures.

## Results

### Mating success, fertility, hatching success and survival of hybrids

During five breeding seasons, the breeding male of the *E*. *angramainyu* was successively paired with 17 virgin females of the *E*. *macularius*. 15 of these females subsequently produced eggs. Since at least one egg of each female hatched, all these females were successfully fertilized by heterospecific matings. The hatchability of the F_1_ hybrids was 44% (n = 70 incubated eggs at 28°C); this value resembles that of the *E*. *angramainyu* (34%, n = 38, 26°C), but is still apparently lower than in *E*. *macularius* (92%, n = 87, 28°C). 25 females and 3 males of 31 F_1_ hatchlings survived to the age of one year (90%). The survival rate was similar to those recorded in the parental species (*E*. *macularius* 84%, *E*. *angramainyu* 85%, n = 80 and 13, respectively). These F_1_ hybrids were further bred to obtain F_2_ and/or B_1_ generations (for hatching success, survival and other details of hybridization experiments, see Table 1).

In order to obtain F_2_ hybrids, three F_1_ hybrid males were consecutively paired with 13 F_1_ hybrid virgin females (six, five and two females with respective males). Each of these 13 females copulated and laid eggs. We incubated 71 eggs (16 eggs from 12 clutches at 26°C and 55 eggs from 29 clutches at 28°C), nevertheless, only four eggs from three different F_1_ hybrid females hatched. All these F_2_ hybrid hatchlings were sired by a single male and incubated at 28°C (hatchability = 6%; no significant effect of temperature on hatchability was detected by Fisher exact test: P = 0.5680). Only one F_2_ hybrid hatchling, a female, survived to the age of one year (Fig 1, see its inborn malformation of the tail). None of the 18 eggs (nine clutches from five females) that were subsequently examined contained a macroscopically visible embryo.

The other 11 F_1_ hybrid virgin females, as well as the six F_1_ females that failed to produce F_2_ or B_1_ hybrids in the previous breeding season were backcrossed with males of the *E*. *angramainyu* or *E*. *macularius*. Ten of them (six virgins) were allowed to copulate with the breeding male of the *E*. *angramainyu*, fertility of which was proved by previous breeding records. Each female laid one egg at least. As in the case of F_2_ hybrids, the eggs were incubated either at 26°C (15 eggs of 10 clutches) or 28°C (16 eggs of 14 clutches). Nevertheless, no juveniles hatched. Moreover, 15 of these eggs (nine clutches from six females) were later dissected and none of them contained a macroscopically visible embryo.

Six of the seven F_1_ hybrid females (five virgin) that copulated with three males of the *E*. *macularius* (three, two and two females, respectively; fertility of these males was proved by previous breeding records) laid eggs and at least five of them were fertile (83%, four of them produced viable offspring, while the remaining fertile female produced just fully developed embryos that failed to hatch). The incubation temperature was randomly set either to 26°C (18 eggs from10 clutches) or 28°C (17 eggs from 9 clutches) and then regularly switched in successive clutches of the female. In a sharp contrast with the negative results of the reverse backcrossing with *E*. *angramainyu* described above, 15 of these 36 eggs hatched (43% hatchability; six hatchlings at 26°C and nine ones at 28°C, no significant effect of temperature on hatchability was detected by the Fisher exact test: P = 0.3145). Three males and eight females survived to the age of one year (73% survival). Additional four dead embryos that failed to hatch (all from 26°C) were found inside 16 dissected eggs belonging to ten clutches produced by five F_1_ hybrid females.

To prove their fertility, 17 F_1_ females that failed to produce F_2_ or B_1_ hybrids in the previous experiments with effect of the incubation temperature were backcrossed again with males of the *E*. *macularius* in the breeding season in 2013. The eggs were incubated at 28°C only. Each female laid at least one egg and 12 of them appeared fertile (71%). Out of the 89 eggs belonging to the 49 clutches, 35 juveniles successfully hatched (hatchability = 39%). 28 of them (80%) survived up to the age of 12 months (one male and 27 females). Taken together with the above data, 16 of the 24 F_1_ hybrid females (67%) were unambiguously fertile.

Ten females of the *E*. *macularius* were allowed to copulate with one of three F_1_ hybrid males (five, two and three females with respective males). Nine of these females produced eggs, 55 eggs were incubated at 28°C and 41 juveniles hatched successfully (75% hatchability); 27 hatchlings (two males and 25 females) survived to adulthood (66% survival).

In order to test the fertility of the B_1_ hybrids, three females MxMA were crossed with a male *E*. *angramainyu*. They produced 17 eggs; 13 eggs were incubated at 28°C and only one juvenile hatched (8%) and survived to the age of one year. Another two females MxMA were crossed with *E*. *macularius* males and they laid seven eggs, six of which were incubated at 28°C and one egg failed. Half of the eggs hatched but only one juvenile survived to adulthood.

To compare the incubation success (hatchability) in paternal species and the available categories of hybrids, we adopted a marginal model (geeglm function, family = binomial, logit link) accounting for an identity of the mother. The model revealed a significant variation of the incubation success among the examined groups (species and categories of hybrids; df = 8, χ^2^ = 76.2, P < 0.0001; Table 2). The incubation success of the *E*. *macularius* was significantly higher than those found in every other examined groups.

**Table 2:** The effects of hybridization on the incubation success (hatchability) of the *E*. *macularius*, *E*. *angramainyu*, and their hybrids. Hybridization crossing - factor group; hatchability - binomial response variable comparing hatched and failed eggs of each clutch. Coefficients (Estimate), its Standard errors (SE), Wald statistics (Wald) and significance of treatment contrasts against reference group *E*. *macularius* (P) are provided. The marginal model (geeglm function, logit link) accounts for a mother’s identity to avoid the problem of pseudoreplications. See Table 1 for explanations of the Generation and Crossing abbreviations.

Generation	Crossing abbreviation	Estimate	SE	Wald	P
	Intercept	2.4178	0.5614	18.55	< 0.0001
**P**_**A**_	A	-3.0041	0.7237	17.23	< 0.0001
**F**_**1**_	MA	-2.6733	0.6359	17.67	< 0.0001
**B**_**1M**_	MxMA	0.4566	1.1785	0.15	0.6984
**B**_**1A**_	MAxA	-5.7987	1.0526	30.35	< 0.0001
**B**_**1M**_	MAxM	-2.732	0.8005	11.65	0.0006
**F**_**2**_	MAxMA	-5.1546	0.8501	36.76	< 0.0001
**B**_**1M**_ **xP**_**A**_	(MxMA)xA	-4.9868	0.8518	34.27	< 0.0001
**B**_**2M**_	(MxMA)xM	-2.4178	0.5614	18.55	< 0.0001

Most of the hatchlings successfully survived up to the age of one year; 84% of *E*. *macularius* (67 of 80), 85% of *E*. *angramainyu* (11 of 13), 90% of F_1_ hybrids (28 of 31) and 72% of pooled categories of F_2_, B_1_ and higher order hybrids (68 of 95). The variation in the survival rate among these groups approached significance (glm, binomial response variable, logit link, χ^2^ = 7.2, df = 3,218, P = 0.0666).

### Morphology of the hybrids

The parental species as well as the F_1_ hybrids exhibit distinct features of a physical appearance including the coloration pattern, body size and shape (for details, see Fig 1). We further examine these traits separately.

#### Coloration pattern

**Fig 3:**
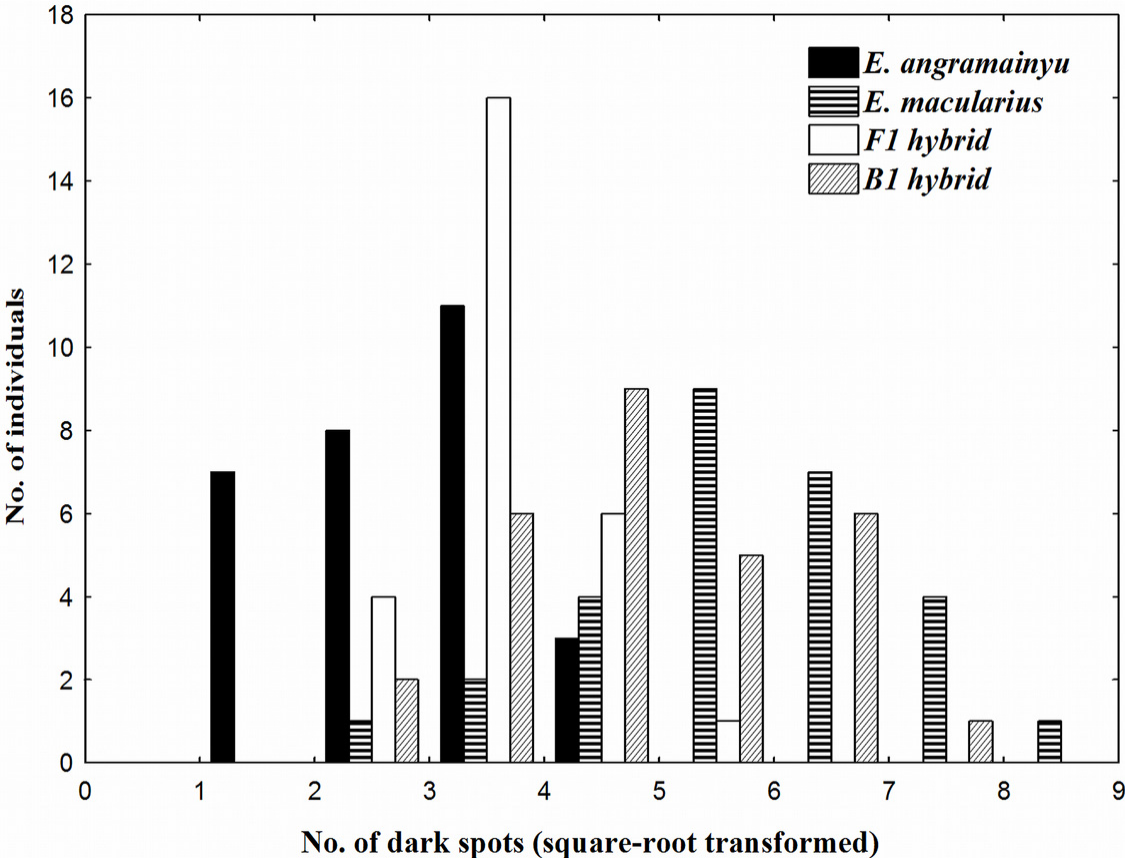
Variation in the number of dark spots on head. *E*. *angramainyu*, *E*. *macularius*, and their F_1_ and B_1M_ hybrids. The number of spots was square-root transformed.

The typical patterns of dark spots on the head of adult individuals differ markedly between the *E*. *angramainyu* and *E*. *macularius*. Large elongated longitudinal spots prevail in the former species, while the presence of numerous, but smaller and rounded spots in the latter one. We examined the number of dark spots as well as the size of the largest one in both parental species and their F_1_ and B_1M_ hybrids (Table 3). ANOVAs revealed a highly significant variation among these groups in both these traits (F_3, 109_ = 38.4, P < 0.0001 and F_3, 107_ = 28.9, P < 0.0001, respectively). The mean values for hybrids were between those of the parental species; F_1_ hybrids were closer to the *E*. *angramainyu* in this respect; the B_1M_ hybrids exhibited values closer to those of the *E*. *macularius* (Fig 3).

**Table 3:** Means and Standard errors (SE) for Number of spots on the head, Spot size and Spot length in the *E*. *angramainyu* (P_A_), *E*. *macularius* (P_M_), and their F_1_ and B_1M_ hybrids. Number of spots on the head—square root transformed, Spot size—area of the largest spot scaled to the head size and natural log-transformed, and Spot length—length of the largest spot scaled to the head length and natural log-transformed. In the case of the Number of spots, post hoc Tukey tests at P < 0.05 were significant for all comparisons. The same procedure revealed two homogenous groups (*E*. *angramainyu* and F_1_; *E*. *macularius* and B_1M_) for the Spot area and Spot length. N–number of animals in the testing group.

Group		Number of spots			Spot size		Spot length	
	N	Mean	SE	N	Mean	SE	Mean	SE
**P**_**A**_	29	2.669	0.203	29	-2.045	0.134	1.192	0.132
**F**_**1**_	28	3.618	0.139	28	-2.254	0.117	0.911	0.111
**B**_**1M**_	27	4.731	0.246	27	-3.208	0.167	0.041	0.149
**P**_**M**_	29	5.767	0.257	27	-3.551	0.123	-0.182	0.104

#### Body size

**Fig 4:**
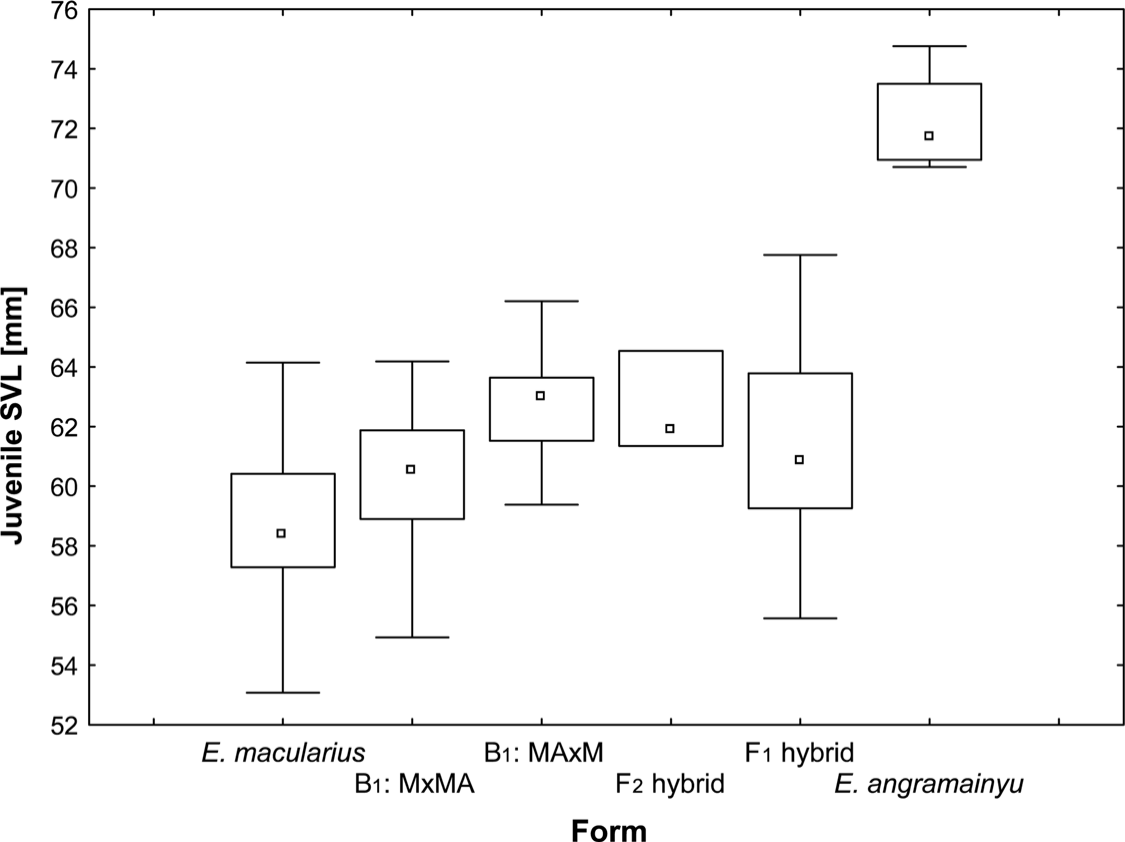
Box plots of hatchling snout-vent lengths. *E*. *macularius* (n = 32), *E*. *angramainyu* (n = 4), their hybrids of the first (F_1_; n = 25) and second (F_2_; n = 3) filial generations and the reciprocal backcrosses of F_1_ males or females to the *E*. *macularius* (B_1M_; the individuals with father F_1_ hybrid are denoted as MxMA, while those with the mother F_1_ hybrid as MAxM; n = 11 and 16, respectively). Median, quartiles and ranges are provided.

**Fig 5:**
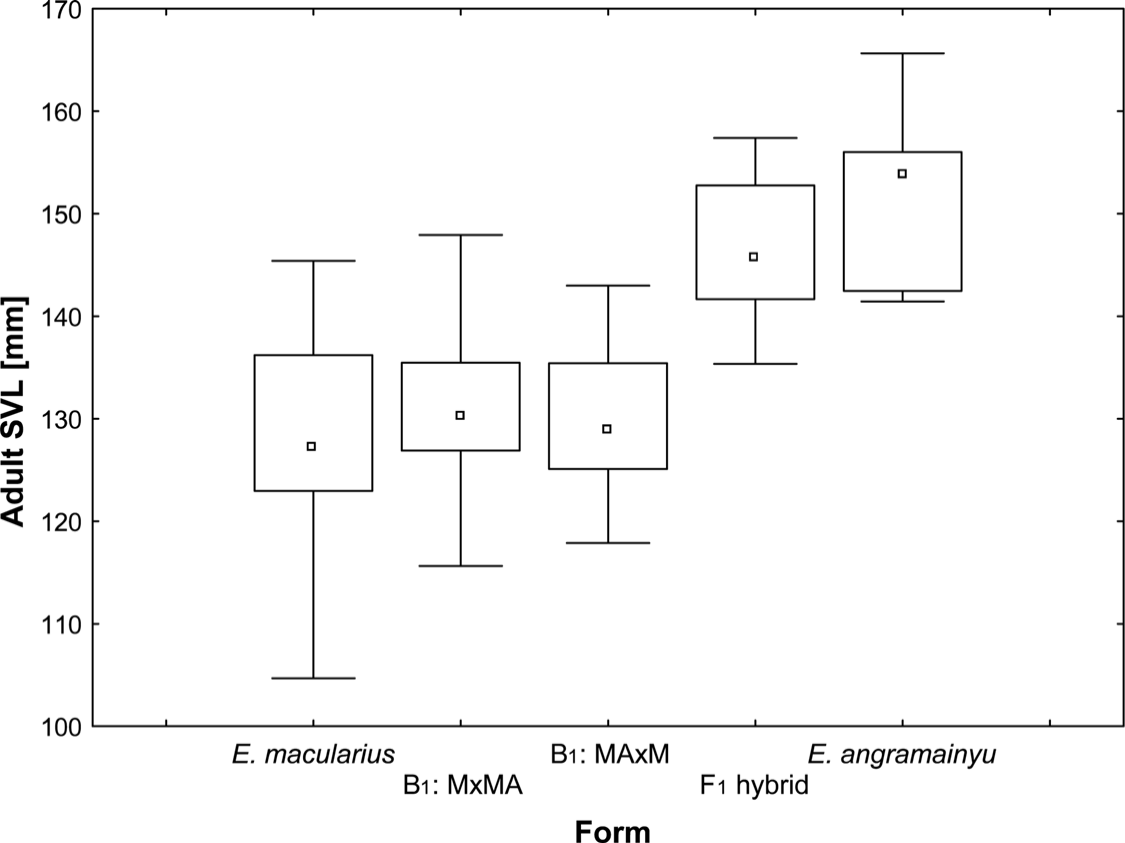
Box plots of adult snout-vent lengths. *E*. *macularius* (n = 68), *E*. *angramainyu* (n = 15), their hybrids of the first filial generation (F_1_; n = 27), and its reciprocal backcrosses of F_1_ males or females to the *E*.*macularius* (B_1M_; the individuals with the father F_1_ hybrid are denoted as MxMA, while those with the mother F_1_ hybrid as MAxM; n = 10 and 17, respectively). Median, quartiles and ranges are provided.

Body size of the *E*. *angramainyu* is considerably larger than in the *E*. *macularius* and this difference is demonstrable both in adults and hatchlings (Figs 4 and 5). Consequently, ANOVAs revealed that the snout-vent length (SVL) varied significantly among of the examined species and their hybrids (F_4, 132_ = 44.05 and F_5, 97_ = 14.42 for adults and hatchlings, respectively; both P < 0.0001). Post hoc tests distinguished two homogenous groups (at α = 0.05; Ps of all significant comparisons are < 0.0001) according to the adult body size; the one containing the *E*. *angramainyu* and F_1_ hybrids, and the other one consisting of the *E*. *macularius* and their B_1M_ hybrids. Also, the body size of the only F_2_ hybrid that survived to adulthood (SVL 129.5mm) was close to the values of the *E*. *macularius*. The corresponding comparisons of the hatchling body size revealed that the *E*. *angramainyu* were larger than the *E*. *macularius* (P = 0.0001) and the hybrids (F_1_, F_2_, both types of B_1M_; Ps: = 0.0002, 0.0029, 0.0008 and 0.0002, respectively). Moreover, the *E*. *macularius* hatchlings were slightly, but significantly smaller than both F_1_ (P = 0.0373) and a specific category of the B_1M_ hybrids (MAxM, i.e., descendants of F_1_ females; P = 0.0115).

#### Body shape

**Fig 6:**
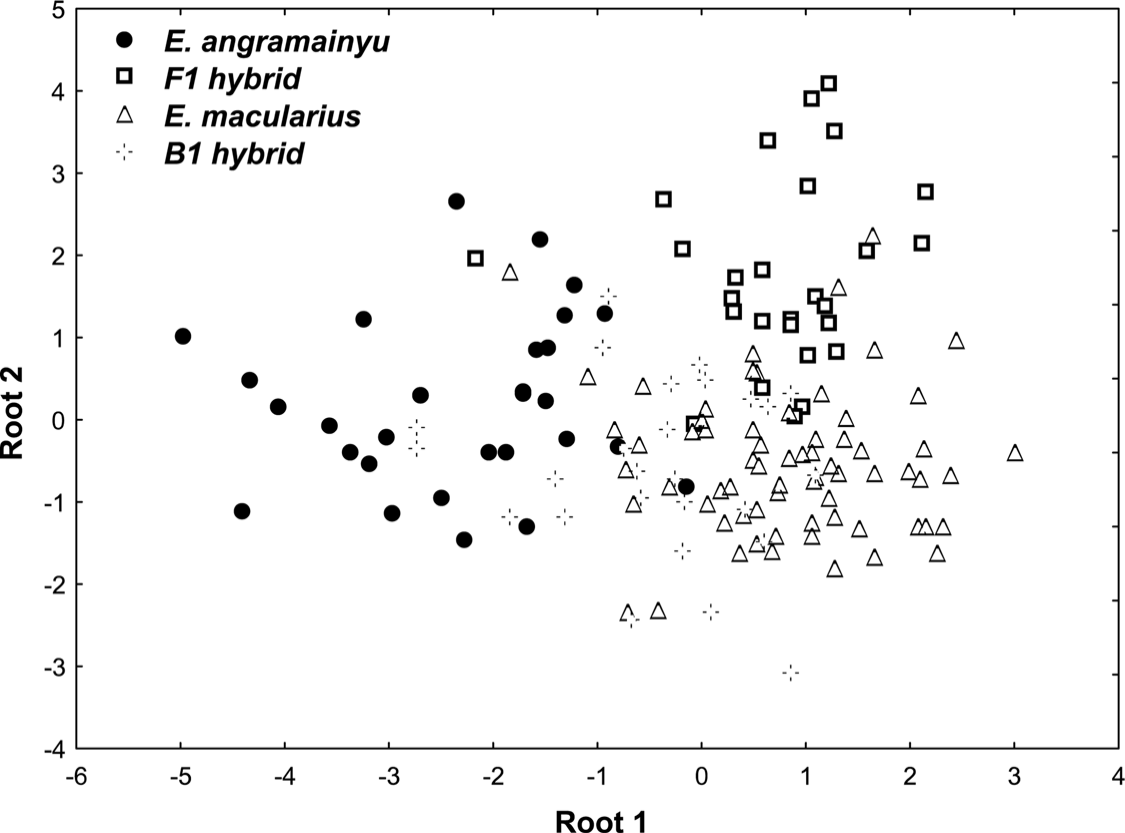
Results of canonical variate analysis extracting multivariate axes (roots 1 and 2). The results discriminated the *E*. *macularius*, *E*. *angramainyu*, and their F_1_ hybrids from 15 size-adjusted morphometric traits. Backcrosses of the F_1_ hybrids with the *E*. *macularius* were also projected into this morphospace.

Canonical variate analysis (CVA) performed on size-adjusted measurements revealed that the body shape differed markedly among the *E*. *macularius*, *E*. *angramainyu* and their F_1_ hybrids (Fig 6). The first canonical axis discriminating the *E*. *macularius* from the *E*. *angramainyu* may be interpreted as a relative length of limbs (the latter species possessing longer limbs; correlations between this axis and limb measurements were: -0.469, -0.353, -0.309, -0.378, and -0.319 for the lengths of femur, tibia, humerus, ulna, and middle finger, respectively), while the second canonical axis discriminating the F_1_ hybrids from the parental species correlated with the snout-vent length (r = 0.594) and head width (r = 0.307). The discriminant function analysis (DFA; Wilks' Lambda = 0.178, F_30, 214_ = 9.76, p < 0.0001) revealed that the overall reclassification success was high (87%), only one of the 29 individuals of the *E*. *angramainyu* and two of the 68 individuals of the *E*. *macularius* were assigned to the opposite species according to their body shape. Out of the 27 F_1_ hybrids, seven were erroneously assigned to the *E*. *macularius* and only one to the *E*. *angramainyu* (see Table 4). Application of the above discriminant functions to the backcrosses and higher order hybrids showed that only one of these animals was classified as an F_1_ hybrid; the others were classified either as the *E*. *macularius* (20 cases) or as the *E*. *angramainyu* (9 cases).

**Table 4:** Results of the discriminant function analysis (DFA) on 15 size-adjusted morphometric traits. The *E*. *macularius*, *E*. *angramainyu*, and their F_1_ hybrids were included in the analysis. Resulting discriminant functions were then applied to the reclassification of these animals as well as additional ones belonging to other categories of their hybrids into these three groups. The numbers indicate assignation of the individual as predicted by DFA. Generation and Crossing abbreviation = see Table 1. No. of examined individuals = the observed number of animals belonging to each category; Reclassification success = percent of individuals assigned to a correct group.

Generation	Crossing abbreviation	Reclassifiction success [%]	P_A_ *E*.*angramainyu*	F_1_ hybrid	P_M_ *E*.*macularius*	No. of examined individuals
**P**_**A**_	A	93	27	1	1	29
**F**_**1**_	MA	70	1	19	7	27
**P**_**M**_	M	91	2	4	62	68
**F**_**2**_	MAxMA	-	0	0	1	1
**B**_**1M**_	MxMA	-	3	0	14	17
**B**_**1M**_	MAxM	-	5	1	4	10
**B**_**2M**_	(MxMA)xM	-	1	0	0	1
**B**_**1M**_ **xP**_**A**_	(MxMA)xA	-	0	0	1	1

## Discussion

### Hybridization success

We demonstrated that the attempts to cross an *E*. *macularius* with an *E*. *angramainyu* regularly result in successful copulations, production of fertilized eggs and well-developed hatchlings. Considering that both geological and genetic evidence suggest that the divergence of the *E*. *macularius* and *E*. *angramainyu* lasted at least 12–15 million years (see under Introduction), even the ability to produce healthy F_1_ hybrids is remarkable. Comparably, divergent species of mammals are typically unable to produce F_1_ hybrids (but see [114, 115]). Thus, our results in the eyelid geckos fit the slow (“avian”) rather than the rapid (“mammalian”) rate of the evolution of postzygotic RIMs [44, 47].

Not the ability to produce F_1_ hybrids, but especially the fertility of the hybrids usually determines the evolutionary consequences of hybridization. Bolonick and Near (2005) [34] demonstrated in centrarchid fishes that the divergence time of species still able to produce fertile hybrids was two times shorted than that of those able to produce viable, but sterile F_1_ hybrids (15 versus 34 million years, respectively, in a similar way in birds [116].

In our experiments, most of the F_1_ hybrids of the *E*. *macularius* and *E*. *angramainyu* appeared fertile when backcrossed with the *E*. *macularius* (see Table 1). Also, at least two from the five resulting backcrosses were fertile. Thus, low success of attempts to produce F_2_ hybrids should be attributed to genetic incompatibility rather than to sterility of the F_1_ hybrids. This conclusion also concerns the failed backcrossing to the *E*. *angramainyu* (see Table 1). In this case, successful copulations initiated laying of eggs, which failed to develop and contained no macroscopically detectable embryos. The likely cause is a defect of either fertilization or early development. The asymmetric pattern of incompatibilities allowing backcrossing of the F_1_ hybrids solely to the *E*. *macularius* is remarkable, but not exceptional. Such asymmetries fit the predictions of some genetic theoreticians [117] and were also previously reported from experiments performed in other animal taxa (e.g., fishes [118], amphibians [119, 120], lizards [69, 121], insects [122]).

Because we have only one breeding male *E*. *angramainyu*, the failed backcrossing to the *E*. *angramainyu* could be due to mating between close relatives, F_1_ hybrid daughters with the *E*. *angramainyu* father, respectively. Similarly, the low success of producing F_2_ hybrids could be determined by the breeding of siblings. On the other hand, the strong inbreeding impact on the fitness in the first generation of the relative breeding in lizards was supported neither by the studies in the literature [123], nor by our own experience with the breeding of closely related animals of the *E*. *macularius*. In case of the Swedish sand lizard, Olsson at al. (2002) demonstrated that the sand lizards produce malformed offspring often when they mate with siblings. However, there is low level of genetic variation and there are monitored similarly malformed offspring in this natural population too (up to 10%) [124]. The higher incidence of the malformed offspring through the mating of siblings is probably the result of inbreeding depression of entire population.

The observed difference in hatching success between the parental species (92% in *E*. *macularius* and 34% in *E*. *angramainyu*) considerably limits the interpretation of the quantitative differences in hatchability between the parental species and their hybrids. The lower hatching success of the *E*. *angramainyu* may be attributed to suboptimal incubation conditions. The optimalization of the incubation temperature of this little known species would need huge number of eggs and would require a separate long-term study. Consequently, it is difficult to distinguish between the additive effects of genes and the effects resulting from the incompatibility of genes originating from different parental species that are present in hybrids. On the other hand, it has not shown that the different optimal incubation temperature of the parental species (26°C or 28°C in this study) would affect the hatchability of the eggs produced by the F_1_ hybrid females.

Due to an extreme rarity of the *E*. *angramainyu*, it was impossible to obtain those combinations of reciprocal crosses involving females of this parental species. Nevertheless, in the genus *Eublepharis*, males are genetically fully equivalent to females due to the presence of temperature sex determination (TSD, [125–127]). This genetic equality of the sexes, however, does not mean an exclusion of the maternal effects and/or sex biased effects of DMIs.

In spite of the difficulties to produce F_2_ hybrids of the *E*. *macularius* and *E*. *angramainyu* and the failed backcrossing of the F_1_ hybrids with the *E*. *angramainyu*, the successful backcrossing of the F_1_ hybrids with the *E*. *macularius* provides a theoretical possibility for introgression of the *E*. *angramainyu* genes into the populations of the *E*. *macularius*. This suggests that postzygotic RIMs between these distinct species have not been completed.

Another aspect of successful hybridization is the viability, developmental stability and health of the hybrids. As repeatedly demonstrated in many model taxa [22, 34, 53, 128, 129], the viability of the F_1_ hybrids may be comparable or even higher than that of the parental species due to the heterosis and the absence of segregation. In contrast, the negative effects of hybridization on post-hatching viability usually result from segregation, and thus, they are confined to the F_2_ generation, backcrosses, and higher order hybrids [21, 130]. In our experiment, the survival rate was high and fairly comparable among the *E*. *angramainyu*, *E*. *macularius*, F_1_ hybrids and the pooled remaining categories of the hybrids. Nevertheless, all four hatchlings belonging to the F_2_ generation showed deformations of the tail suggesting developmental problems during embryogenesis and only one of them survived up to the age of one year. Although the sample size of the F_2_ generation was too small to allow for correct comparison of the survival rate, this record is noticeable.

The presence of TSD in the genus *Eublepharis* [125–127], which complicates the evolution of functionally differentiated sex chromosomes [131], may provide an alternative explanation of the geckos’ ability to produce fertile between-species hybrids. In many animal taxa with genetic sex determination (GSD), fitness of the hybrids is strongly sex-biased. Following the empirical Haldane’s rule [132], hybrids of a heterogametic sex are regularly more affected by incompatibilities and consequent infertility. The genes responsible for the speciation (DMIs) tend to be recessive and localized on the non-homological part of the X or Z chromosomes (the sex chromosomes present in a homogametic sex; [31, 133]). Thus, it may be expected that the absence of sex chromosomes retard the evolution of the postzygotic reproductive isolating mechanisms (RIMs). Nevertheless, the list of the reptilian taxa, in which the hybridization among distant species was reported, contains not only clades with the TSD (chelonians, crocodylians), but also many species with the GSD (e.g., iguanids [62–65] and colubrid snakes [75, 134, 135]; for evolution of sex determination mechanisms among squamates see [136]). Surprisingly, a recent review of hybridization events in lizards showed that reliable reports about hybridization of species with TSD are lacking [44]. In this context, the fact that the HKY distance of the mt cyt b gene sequences of the *E*. *angramainyu* and *E*. *macularius* (22%) is higher than those in all other pairs of hybridizing lizard species reported to date [44].

Published studies properly documenting experimental hybridization of distinct lizard species are extremely scarce [69, 70, 137–140], for review see [44]. There is, however, a study performed in a model system of European lizards with GSD exhibiting a degree of genetic differentiation [141], which is roughly comparable to the one occurring between the *E*. *macularius* and *E*. *angramainyu* possessing TSD. Rykena (1991, 1996, 2002) [67–69] performed experimental crossings among five species belonging to the genus *Lacerta* (*L*. *viridis*, *L*. *agilis*, *L*. *strigata*, *L*. *schreiberi*, and *L*. *trilineata*) with well-differentiated sex chromosomes (ZW). The author confirmed a sex bias predicted by the Haldane’s rule, i.e., the hybrid infertility affected the heterogametic females, but not the homogametic males of between-species hybrids. The rate of female infertility proved by both breeding and dissection of the reproductive organs varied among pairs of the hybridized species. The attempts to produce F_1_ hybrids and backcrosses (via fertile male hybrids) were repeatedly successful, while the F_2_ hybrids were only rare. Thus, these thorough experiments demonstrated that a gene flow among the studied species of the genus *Lacerta* is not entirely precluded by postzygotic RIMs in spite of GSD. Consequently, to properly answer the question whether the TSD enhances the success of hybridization between distinct species, additional experimental data are required. Multiple pairs of either TSD or GSD species with similar divergence time need to be crossed and the efficiency of the recorded RIMs compared.

### Phenotype of the hybrids

Our morphological analyses confirmed a clear differentiation of the studied populations of the *E*. *macularius* and *E*. *angramainyu* in the body size and shape, as well as in the coloration pattern. The phenotype of the descendants of the *E*. *macularius* mothers sired by *E*. *angramainyu* (or F_1_ hybrid) males contained clear paternal characters. This excludes the theoretical possibility of their parthenogenetic origin instead of hybridization. It is in accord with the absence of any record of parthenogenesis in the family Eublepharidae (for recent records of parthenogenesis in other reptiles, see [142–147]).

As expected, hybrid specimens tend to show intermediate characters, but a resemblance of the hybrid phenotype to the paternal and maternal ones varies among crossings and differs from a trait to a trait. The F_1_ hybrids, descendants of an *E*. *macularius* female and an *E*. *angramainyu* male, resemble the *E*. *angramainyu* in their large adult body size, which strongly contrasts with a small body size of the hatchlings (which is close to that of their mothers). This may be interpreted either as dominance of the paternal alleles or as a result of enhanced growth enabled by the heterosis. In contrast, body shape of the F_1_ hybrids was close to that of the *E*. *macularius* along the first canonical axis (CV1; short limbs), but showed a specific feature (longer SVL and wider head) that differed from both the paternal species as well as the higher order hybrids on the CV2 axis (see Fig 6).

Similar unique characters of hybrids were demonstrated in other taxa like transgression segregation [18, 148–150]. These novelties may be preferred in some ecological conditions (e. g., suboptimal for parent species [22]). In some cases, the hybrids were reported to be possibly more competitive than the parent species (e.g., parthenogenetic species [151], but see [6, 22]). It is known that certain body constitution is optimal for a specific habitat (grassland, rocky land, sand dunes) and is also positively selected for different mobility. Long legs are better for sprint and jumping, short robust legs are favored for burrowing and rock climbing [152–154]. Due to the origin of the transgression characters or intermediate characters of hybrids, these specimens could occupy new ecological niches [155], gain new food sources [22], be better in some performance activities [148, 156], and then be more successful against predators or in male fights over territories and mating rights than one or both of the parental species. Nevertheless, relatively instantaneous combination of traits developed due to hybridization facilitates a rapid adaptive radiation [4, 157] and offers fresh evolutionary scenarios for re-examination in nature selection.

## Conclusions

We demonstrated that the *E*. *macularius* is able to hybridize with its congeneric species, the *E*. *angramainyu*. F_1_ hybrids are viable and fertile, and introgression of the *E*. *angramainyu* genes into the *E*. *macularius* genome is enabled via backcrossing. The examined hybrids (except those of the F_2_ generation) displayed neither malformations nor reduced survival. Analyses of morphometric and coloration traits confirmed phenotypic distinctness of both parental species and their F_1_ hybrids.

These findings contrast with the scenario of a long-term geographic and evolutionary separation of these species, which is supported by both biogeographic and genetic arguments.

In conclusion, occurrence of fertile hybrids of distinct species, which are comparably divergent such as the *E*. *angramainyu* and *E*. *macularius*, may be also expected in other taxa of squamates. This would violate the current estimates of species diversity in lizards as well as warn against taxonomic decisions leading to excessive splitting of lizard species.
